# Metformin Use and Mortality in Women with Ovarian Cancer: An Updated Meta-Analysis

**DOI:** 10.1155/2022/9592969

**Published:** 2022-02-28

**Authors:** Mingchuan Guo, Xiaofei Shang, Duanying Guo

**Affiliations:** ^1^Department of Gynecology, Longgang District People's Hospital of Shenzhen, Shenzhen 518172, China; ^2^Department of Physical Examination, Cancer Hospital of Chinese Academy of Medical Sciences, Shenzhen Center, Shenzhen 518116, China

## Abstract

**Background:**

Previous observational studies and meta-analysis suggested a possible association between metformin use and reduced mortality in women with ovarian cancer (OC). However, clinical factors that may influence the relationship remain poorly evaluated. We performed an updated meta-analysis to systematically evaluate the above association and to observe the potential influences of study characteristics on the association.

**Methods:**

Relevant studies reporting the association between metformin use and mortality in women with OC in the multivariate adjusted model were identified by search of electronic databases that included PubMed, Embase, and Web of Science. The random-effects model was adopted to combine the results.

**Results:**

Nine studies including 10030 women with OC were included. Overall, metformin use was independently associated with reduced overall mortality (hazard ratio (HR): 0.72, 95% confidence interval (CI): 0.55–0.93, *P*=0.01; *I*^2^ = 62%). Consistent results were observed for studies comparing metformin users with nondiabetic women and studies comparing metformin users with diabetic women who did not use metformin (*P* for subgroup analysis = 0.70). Further subgroup analyses showed consistent results in studies with metformin use before or after the diagnosis of OC, with or without adjustment of body mass index (BMI) and with or without adjustment of concurrent medications (*P* for subgroup analyses all >0.10).

**Conclusion:**

Metformin use is associated with reduced mortality in women with OC, which may be independent of the diabetic status of the controls, timing of metformin use, or adjustment of BMI and concurrent medications. Clinical trials are needed to validate the potential benefits of metformin on survival of OC.

## 1. Introduction

Ovarian cancer (OC) is the fifth leading cause of cancer-related mortality globally, among which epithelial OC is the most common type with a high mortality [1]. Although OC is relative rare among gynecological malignancies, women with OC are usually diagnosed late due to the nonspecific symptoms and lack of the screening test for the disease [2–4]. Surgical resection is preferable for women with early stage OC; while for most cases of advanced cancer, tumor debulking followed by adjunctive therapy could be performed. However, the recurrence of the cancer remains high despite of these treatments [5–7]. Therefore, effective treatments are urgently needed to improve the survival and quality of life in women with OC. Metformin is a conventional oral antidiabetic agent which has been suggested to confer anticancer efficacy [8]. Previous studies have confirmed that metformin use is associated with reduced risk of cancer in diabetic patients, including the incidence of OC [9–11]. However, studies evaluating the influence of metformin on mortality in women with OC showed inconsistent results [12–20]. Some studies suggested that metformin use was associated with reduced mortality in women with OC [12–14, 18, 20], while others did not [15–17, 19]. Accordingly, some meta-analyses have been performed to evaluate the association between metformin use and mortality in OC [21–24]. Although most of their findings suggested that a possible relationship between metformin use is associated with reduced mortality in women with OC, potential influences of patient or study characteristics on the association have rarely been observed in previous meta-analyses, such as diabetic status of the women in the control group, timing of metformin use, and obesity status of the patients. Understanding the possible influences of these clinical variables on the association between metformin use and reduced mortality in women with OC is important for designing future clinical studies. Therefore, in this study, we performed an updated meta-analysis to provide a current understanding for the association between metformin use and mortality in women OC. In addition, comprehensive sensitivity and subgroup analyses were performed to evaluate whether the abovementioned study or patient characteristics may affect the association of interest.

## 2. Methods

The Meta-analysis of Observational Studies in Epidemiology (MOOSE) guideline [25] and Cochrane's Handbook [26] were followed in this study.

### 2.1. Literature Search

The electronic databases of PubMed, Embase, and Web of Science databases were searched with a strategy of combined terms: “metformin;” “ovarian” OR “ovary;” “cancer” OR “carcinoma” OR “tumor” OR “malignancy” OR “neoplasm;” and “death” OR “deaths” OR “mortality” OR “survival” OR “prognosis.” Only studies reported in English were considered. References of related articles or reviews were also analyzed. The final literature search was performed on March 23, 2021.

### 2.2. Study Identification

Studies fulfilled these criteria were included in the meta-analysis: longitudinal follow-up studies (cohort studies and nested case-control studies) published as full-length papers; included women with confirmed diagnosis of OC; compared the mortality between users and nonusers of metformin during follow-up; and reported hazard ratios (HRs) for the association between metformin use and all-cause mortality with multivariate analysis. Definition of metformin use was consistent with that applied among the included studies. Reviews, preclinical studies, cross-sectional studies, and irrelevant studies were not included.

### 2.3. Data Extracting and Quality Evaluation

Two authors implemented database search, data extraction, and study quality assessment separately. If disagreements occurred, they were discussed with the corresponding author. The following data were recorded: author and study year; study design characteristics and country of the study; sample size of the included women with OC, mean age of the women, and International Federation of Gynecology and Obstetrics (FIGO) stage of OC; definition of metformin use; median follow-up duration and methods for validation of mortality; and potential confounding factors adjusted in the multivariate analyses. The Newcastle–Ottawa Scale [27] was used evaluate the quality of the included studies. This scale rated from 1 to 9 stars and reflected the quality of the study by aspects of participant selection, comparability between groups, and outcome validation.

### 2.4. Statistical Analyses

HRs and the corresponding 95% confidence intervals (CIs) were extracted for each included study. For studies reporting multiple HRs according to different models of multivariate regression analyses, the most adequately adjusted HR from each study was extracted and combined in this meta-analysis. Then, standard errors (SEs) of the logarithmic transformation of the HRs were estimated from the 95% CIs or *P* values. For normalization of their distribution, HRs were logarithmically transformed [26] and combined. Heterogeneity within the included cohort studies was tested via Cochrane's *Q* test, as well as the estimation of *I*^2^ statistic [28]. An *I*^2^ > 50% suggests significant heterogeneity. A random-effects model was chosen to combine HRs by incorporating the potential heterogeneity between studies [26]. Sensitivity analyses by sequentially excluding each dataset at a time (influencing analyses) were conducted to clarify the influence of a certain study on the overall results [29]. Predefined subgroup analyses according to the diabetic status of women in the control group, timing of metformin use, and adjustment of body mass index (BMI) or concurrent medications were also performed. Visual examination of the symmetry of the funnel plots were used for the assessment of publication bias [30], which were further validated by Egger's regression asymmetry test. The RevMan (Version 5.1; Cochrane Collaboration, Oxford, UK) and Stata software were involved for statistical analyses.

## 3. Results

### 3.1. Database Search

**Figure 1:**
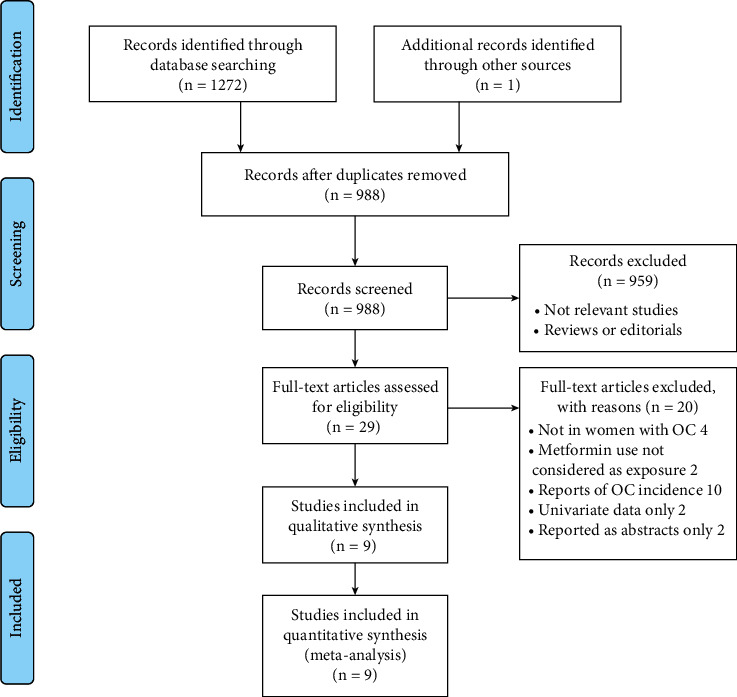
Scheme of study inclusion following PRISMA.

Details of the database search are shown in Figure 1. The first-step database search retrieved 988 articles after duplicated studies were excluded. Among them, 959 studies were further excluded based on titles and abstracts primarily because they were not related to the purpose of the meta-analysis. Then, for the remaining 29 studies evaluated by full-text reading, 20 were further excluded for the reasons shown in Figure 1, which resulted in 9 studies finally analyzed in the meta-analysis [12–20].

### 3.2. Study Characteristics

Characteristics of each study of the meta-analysis are given in Table 1. Overall, 9 longitudinal follow-up studies including 10030 women with OC were considered eligible for the meta-analysis [12–20]. All of these studies were of retrospective design and published between 2012 and 2020. These studies were performed in the United States [13–15, 17, 20], United Kingdom [12], Finland [19], Israel [16], and China [18], respectively. Since one study [18] reported data in patients with continuous and discontinued metformin use separately, these two datasets were included independently in the current meta-analysis. Six studies included women with OC without restriction of pathological type [12, 14, 16–19], while the other 3 included women with epithelial OC [13, 15, 20]. The sample sizes of the included studies varied from 143 to 5126, and the mean ages of the included women varied between 57 and 73 years. Use of metformin was generally evidenced by the records of medical or pharmacy database. The median follow-up durations varied from 2.1 to 7.2 years, and outcome of mortality was validated by records of medical database among the included studies. Potential confounding factors including age, BMI, tumor stage and histological type, anticancer treatment, comorbidities, and concurrent medications were generally adjusted to a varying degree in the multivariate analyses for the association between metformin use and mortality in OC. The quality of these studies was good, evidenced by 7–9 points of NOS scores (Table 2).

### 3.3. Association between Metformin Use and Mortality Risk in Women with OC

**Figure 2:**
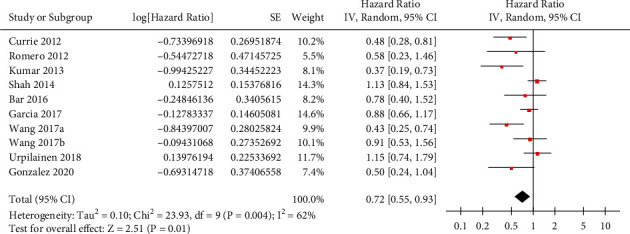
Forest plots for the overall meta-analysis of the association between metformin use and all-cause mortality in women with OC.

**Figure 3:**
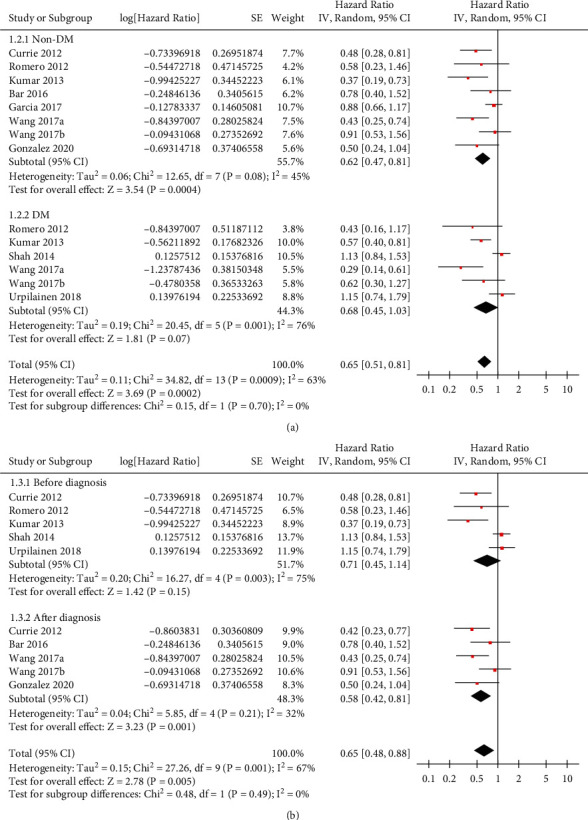
Forest plots for the subgroup analysis of the association between metformin use and all-cause mortality in women with OC. (a) Subgroup analysis according to the diabetic status of the women in the control group. (b) Subgroup analysis according to the timing of metformin use.

**Figure 4:**
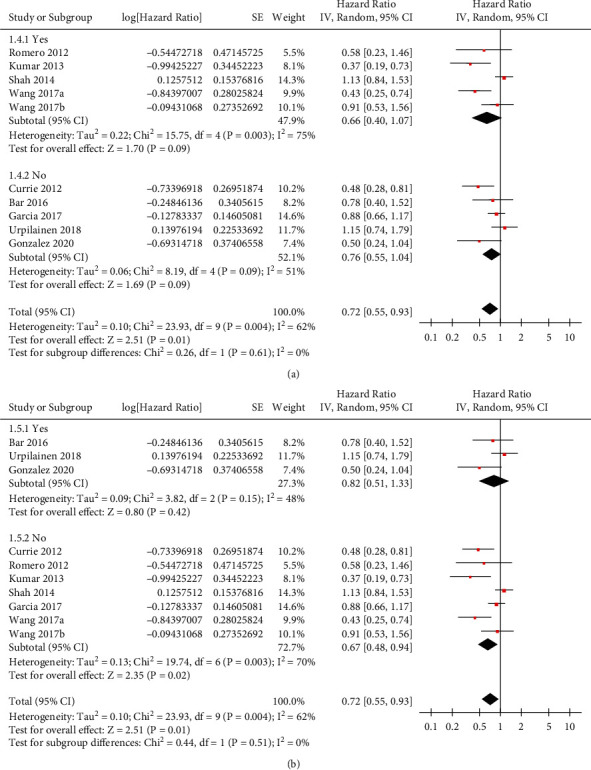
Forest plots for the subgroup analysis of the association between metformin use and all-cause mortality in women with OC. (a) Subgroup analysis according to the adjustment of BMI in the multivariate model. (b) Subgroup analysis according to the adjustment of concurrent medications in the multivariate model.

Moderate heterogeneity was detected among the included retrospective studies (*P* for Cochrane's *Q* test = 0.004, *I*^2^ = 62%). Pooled results of the 9 studies [12–20] with a random-effects model showed that compared to nonmetformin users with OC, metformin use was independently associated with significantly reduced risk of all-cause mortality (adjusted HR: 0.72, 95% CI: 0.55–0.93, *P*=0.01; Figure 2). Influencing analyses showed consistent results after omitting one dataset at a time (HR: 0.73–0.85, *P* all <0.05). Particularly, sensitivity analysis by excluding the dataset of Wang 2017a or Wang 2017b showed a consistent result (HR = 0.77 and 0.69, respectively, both *P* < 0.05). Subgroup analyses according to the diabetic status of the women in the control group showed consistent results in studies comparing metformin users with nondiabetic women and in studies comparing metformin users with diabetic women who did not use metformin (*P* for subgroup analysis = 0.70; Figure 3(a)). In addition, subgroup analyses showed that timing of metformin use (before versus after the diagnosis of OC) did not significantly affect the association between metformin use and reduced mortality risk in OC (*P* for subgroup analysis = 0.49; Figure 3(b)). Finally, subgroup analyses showed consistent results in studies with or without adjustment BMI (*P* for subgroup analysis = 0.61; Figure 4(a)) and in those with or without adjustment of concurrent medications (*P* for subgroup analysis = 0.51; Figure 4(b)).

### 3.4. Publication Bias

**Figure 5:**
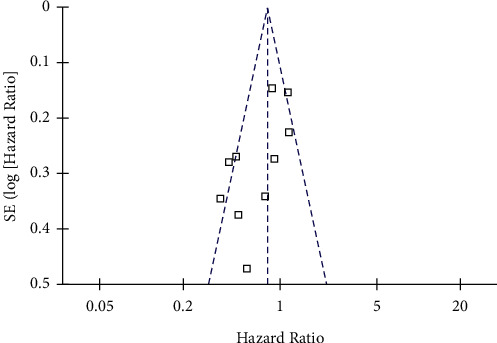
Funnel plots for the meta-analysis of the association between metformin use and all-cause mortality in women with OC.

Funnel plots representing the meta-analysis of metformin use and mortality outcome in women with OC are shown in Figure 5, which are symmetrical, suggesting low risk of publication bias. Egger's regression test showed consistent result for OS (*P*=0.332) (Figure 5).

## 4. Discussion

In this updated meta-analysis, we combined the results of 9 retrospective longitudinal follow-up studies and showed that metformin use is associated with reduced all-cause mortality in women with OC. Sensitivity analysis by excluding one study at a time confirmed the robustness of the finding, which was not primarily driven by either of the included studies. Different from previous meta-analyses, we further performed multiple predefined subgroup analyses, which showed that the potential benefits of metformin on survival were independent of the diabetic status of control women, timing of metformin use, BMI of the participants, and concurrent medications used. Taken together, results of this meta-analysis suggest that current evidence from retrospective studies supports that metformin use is associated with reduced risk of mortality in women with OC. Clinical trials should be performed to evaluate the potential benefits of additional metformin use on survival in women with OC.

Several meta-analyses have been previously published to evaluate the association between metformin and morality in women with OC [21–24]. However, results of these meta-analyses remain inconsistent, and the influence of metformin on mortality in women OC is still not determined. Our updated meta-analysis has the following strengths compared to the previous ones [21–24]. First, only studies with multivariate analysis were included, aiming to provide an independent association between metformin use and reduced mortality in women with OC. Second, only studies published as full-text article in peer-reviewed journals were included, which may avoid the potential bias by including conference abstracts that may not strictly peer reviewed. Third, updated literature search was performed, and 10 datasets from 9 studies including 10030 women with OC were included. Finally, this relatively large sample size of available datasets enabled us to perform multiple predefined subgroup analyses, which were rarely performed in previous meta-analyses. Taken together, results of our meta-analysis supported that metformin use may be associated with reduced mortality in women with OC. Accumulating evidence from basic research studies showed various mechanisms underlying the potential anticancer efficacy of metformin in OC, such as modulating the immunological and/or anti-inflammatory responses, reducing proliferation of cancers, limiting the cancer cell's metabolic plasticity, inhibiting cancer cell migration, reversing chemoresistance, and avoiding epithelial mesenchymal transition [31], which are consistent with the findings of the meta-analysis which showed additional benefits of metformin on survival in women with OC. These findings highlight the importance of performing clinical trials to evaluate the influence of metformin on survival in women with OC.

Interpretation of the results of subgroup analysis may be important for designing clinical trials evaluating the influence of metformin use on survival in women with OC. For example, previous meta-analyses mostly simply compared the mortality in users and nonusers of metformin with OC, regardless of the diabetic status of the women in the control group [21–24]. However, the diabetic status of the women in the control group may affect the findings since diabetes itself has been recognized as a risk factor for worse survival in women with OC [32]. Our subgroup analysis showed that users of metformin had reduced mortality compared to nondiabetic controls and compared to diabetic controls that did not use metformin, which further confirmed the benefits of metformin on survival in women with OC. In addition, obesity has been related with increased risk of mortality in women with OC [33, 34], while metformin use has been associated with reduced BMI [35]. Therefore, it should be determined whether the benefits of metformin on survival in OC are dependent on the role of metformin for reducing BMI. Our subgroup results showed a consistent association between metformin and reduced mortality in women with OC in studies with or without adjustment of BMI. These findings are consistent with the findings of experimental studies which suggested various mechanisms underlying the potential anticancer efficacy of metformin [36, 37]. Also, patients using metformin are also likely to have multiple metabolic comorbidities which require other concurrent medications, such as aspirin and statins. However, using these medications has been also associated with reduced mortality in women with OC [38, 39]. Therefore, it is important to determine that the benefit of metformin on survival in women with OC is independent of the possible influences of concurrent medications. This is again supported by the results of our subgroup analysis which showed a consistent association between metformin and reduced mortality in women with OC in studies with or without adjustment of using concurrent medications.

This study also has limitations. First, all of the included studies in the meta-analysis were retrospective studies, results of which may be affected by selection bias. Prospective studies, with adequate sample size and consecutively included women with OC, are needed to confirm our findings. Besides, clinical trials evaluating the possible survival benefit of metformin use in women with confirmed diagnosis of OC should also be considered. Moreover, this meta-analysis was based on data of the study level but not from individual patients, which prevented further analyses on the influence of patient characteristics on the outcome, such as duration of diabetes, status of glycemic control, and pathological type of OC. Besides, although multivariate adjusted HR was used, we could not exclude the possibility of residual factors which may confound the association between metformin use and reduced mortality. Also, including only studies reporting adjusted association estimates may lead to the results less affected by confounding compared to those based also on crude estimates, while including only studies with multivariate analysis may also lead to a selection of only high-quality studies and a subsequent risk of publication bias. However, no significant bias was detected in the visual examination of the funnel plots or according to the result of Egger's regression test. In addition, two datasets of cohorts that shared the same control group were included in the meta-analysis (Wang 2017a and Wang 2017b), which may influence the variability of the pooled estimates. However, sensitivity analysis by excluding the dataset of Wang 2017a or Wang 2017b also showed consistent result. Finally, a dose-response relationship or a causative association between metformin and reduced mortality in women with OC could not be determined based our meta-analysis. Large-scale clinical trials are needed for further evaluation.

In conclusion, this meta-analysis showed that current evidence from retrospective studies supports that metformin use is associated with reduced risk of mortality in women with OC. The association may be independent of the diabetic status of the women in the control group and BMI and concurrent medications of the patients. Clinical trials are needed to validate the potential benefits of additional metformin use on survival in women with OC.

## Figures and Tables

**Table 1:** Characteristics of the included studies.

Study	Design	Country	Patient characteristics	Patient number	Mean age (years)	FIGO stage	Definition of metformin use	Median follow-up durations (years)	Mortality validation	Variables adjusted

Currie, 2012	R	UK	Women with OC	5126	67.5	NR	Metformin use 30 days before or after OC diagnosis by pharmacy records	2.1	Medical database	Age, smoking history, Townsend index of deprivation, CCI, number of primary care contacts, and year of diagnosis
Romero, 2012	R	USA	Women with epithelial OC	341	59.2	I–IV	Metformin use 30 days before OC diagnosis	5.3	Medical database	Age, BMI, creatinine, histological subtype, grade, and FIGO stage of the tumor
Kumar, 2013	R	USA	Women with OC	239	60.4	I–IV	Metformin use before OC diagnosis by pharmacy records	4	Medical database	Age, BMI, grade, histology, and chemotherapy
Shah, 2014	R	USA	Women with epithelial OC	367	63.5	I–IV	Metformin use before OC diagnosis by medical records	4.8	Medical database	Age, stage, grade, histology, debulking status, BMI, and chemotherapy
Bar, 2016	R	Israel	Women with OC	143	62.5	I–IV	Metformin use after OC diagnosis by pharmacy records	4.1	Medical database	Age, stage, use of neoadjuvant chemotherapy, the presence of DM, HTN, and concurrent medications
Garcia, 2017	R	USA	Women with OC	2291	73	I–IV	Metformin use 6 months before or after OC diagnosis by pharmacy records	5	Medical database	Age, race, diagnosis year, stage, histology, grade, DM, and CCI
Wang, 2017	R	China	Women with OC	568	57.9	I–IV	Metformin use after OC diagnosis by pharmacy records	4.9	Medical database	Age, histological subtype, grade, BMI, smoking, type of surgery, postoperative residual disease, and chemotherapy
Urpilainen, 2018	R	Finland	Women with T2DM and OC	421	71	I–IV	Metformin use before OC diagnosis by pharmacy records	7.2	Medical database	Age, diagnosis year, duration of DM, stage, and concurrent medications
Gonzalez, 2020	R	USA	Women with stage IIIC and IV epithelial OC	534	64	III-IV	Metformin use after OC diagnosis by pharmacy records	4.8	Medical database	Age, race, CCI, stage, chemotherapy, histology, residual disease status, and concurrent medications

FIGO, International Federation of Gynecology and Obstetrics; R, retrospective; OC, ovarian cancer; NR, not reported; CCI, Charlson comorbidity index; BMI, body mass index; DM, diabetes mellitus; HTN, hypertension.

**Table 2:** Detailed quality evaluation for the included studies with the Newcastle–Ottawa scale.

Study	Representativeness of the exposed cohort	Selection of the nonexposed cohort	Ascertainment of exposure	Outcome not present at baseline	Control for age	Control for other confounding factors	Assessment of outcome	Enough long follow-up duration	Adequacy of follow-up of cohorts	Total

Currie, 2012	1	0	1	1	1	1	1	0	1	7
Romero, 2012	1	1	1	1	1	1	1	1	1	9
Kumar, 2013	1	1	1	1	1	1	1	1	1	9
Shah, 2014	1	1	1	1	1	1	1	1	1	9
Bar, 2016	1	0	1	1	1	1	1	1	1	8
Garcia, 2017	1	0	1	1	1	1	1	1	1	8
Wang, 2017	1	1	1	1	1	1	1	1	1	9
Urpilainen, 2018	1	1	1	1	1	0	1	1	1	8
Gonzalez, 2020	0	0	1	1	1	1	1	1	1	7

## Data Availability

The data used to support the findings of this study are available from the corresponding author upon request.

## References

[1] Lheureux S., Gourley C., Vergote I., Oza A. M. (2019). Epithelial ovarian cancer. *The Lancet*.

[2] Sessa C., Schneider D. T., Planchamp F. (2020). ESGO-SIOPE guidelines for the management of adolescents and young adults with non-epithelial ovarian cancers. *The Lancet Oncology*.

[3] Hurwitz L. M., Pinsky P. F., Trabert B. (2021). General population screening for ovarian cancer. *Lancet*.

[4] Wang E. W., Wei C. H., Liu S. (2020). Frontline management of epithelial ovarian cancer-combining clinical expertise with community practice collaboration and cutting-edge research. *Journal of Clinical Medicine*.

[5] Conte C., Fagotti A., Avesani G. (2021). Update on the secondary cytoreduction in platinum-sensitive recurrent ovarian cancer: a narrative review. *Annals of Translational Medicine*.

[6] Song Y. J. (2021). Prediction of optimal debulking surgery in ovarian cancer. *Gland Surgery*.

[7] Baert T., Ferrero A., Sehouli J. (2021). The systemic treatment of recurrent ovarian cancer revisited. *Annals of Oncology*.

[8] Urpilainen E., Puistola U., Boussios S., Karihtala P. (2020). Metformin and ovarian cancer: the evidence. *Annals of Translational Medicine*.

[9] Tseng C.-H. (2015). Metformin reduces ovarian cancer risk in Taiwanese women with type 2 diabetes mellitus. *Diabetes*.

[10] Wen Q., Zhao Z., Wen J. (2019). The association between metformin therapy and risk of gynecological cancer in patients: two meta-analyses. *European Journal of Obstetrics & Gynecology and Reproductive Biology*.

[11] Mekuria A. N., Ayele Y., Tola A., Mishore K. M. (2019). Monotherapy with metformin versus sulfonylureas and risk of cancer in type 2 diabetic patients: a systematic review and meta-analysis. *Journal of Diabetes Research*.

[12] Currie C. J., Poole C. D., Jenkins-Jones S., Gale E. A. M., Johnson J. A., Morgan C. L. (2012). Mortality after incident cancer in people with and without type 2 diabetes: impact of metformin on survival. *Diabetes Care*.

[13] Romero I. L., McCormick A., McEwen K. A. (2012). Relationship of type II diabetes and metformin use to ovarian cancer progression, survival, and chemosensitivity. *Obstetrics & Gynecology*.

[14] Kumar S., Meuter A., Thapa P. (2013). Metformin intake is associated with better survival in ovarian cancer: a case-control study. *Cancer*.

[15] Shah M. M., Erickson B. K., Matin T. (2014). Diabetes mellitus and ovarian cancer: more complex than just increasing risk. *Gynecologic Oncology*.

[16] Bar D., Lavie O., Stein N., Feferkorn I., Shai A. (2016). The effect of metabolic comorbidities and commonly used drugs on the prognosis of patients with ovarian cancer. *European Journal of Obstetrics & Gynecology and Reproductive Biology*.

[17] Garcia C., Yao A., Camacho F., Balkrishnan R., Cantrell L. A. (2017). A SEER-Medicare analysis of the impact of metformin on overall survival in ovarian cancer. *Gynecologic Oncology*.

[18] Wang S.-B., Lei K.-J., Liu J.-P., Jia Y.-M. (2017). Continuous use of metformin can improve survival in type 2 diabetic patients with ovarian cancer: a retrospective study. *Medicine (Baltimore)*.

[19] Urpilainen E., Marttila M., Hautakoski A. (2018). Prognosis of ovarian cancer in women with type 2 diabetes using metformin and other forms of antidiabetic medication or statins: a retrospective cohort study. *BMC Cancer*.

[20] Gonzalez R., Gockley A. A., Melamed A. (2020). Multivariable analysis of association of beta-blocker use and survival in advanced ovarian cancer. *Gynecologic Oncology*.

[21] Wang Y., Liu X., Yan P., Bi Y., Liu Y., Zhang Z.-J. (2019). No effect of metformin on ovarian cancer survival: a systematic review and meta-analysis of cohort studies. *Current Pharmaceutical Design*.

[22] Gong T.-T., Wu Q.-J., Lin B., Ruan S.-K., Kushima M., Takimoto M. (2019). Observational studies on the association between post-diagnostic metformin use and survival in ovarian cancer: a systematic review and meta-analysis. *Frontiers in Oncology*.

[23] Shi J., Liu B., Wang H., Zhang T., Yang L. (2019). Association of metformin use with ovarian cancer incidence and prognosis: a systematic review and meta-analysis. *International Journal of Gynecological Cancer*.

[24] Lu M. Z., Li D. Y., Wang X. F. (2019). Effect of metformin use on the risk and prognosis of ovarian cancer: an updated systematic review and meta-analysis. *Panminerva Medica*.

[25] Stroup D. F., Berlin J. A., Morton S. C. (2000). Meta-analysis of observational studies in epidemiology: a proposal for reporting. Meta-analysis of Observational Studies in Epidemiology (MOOSE) group. *JAMA*.

[26] Higgins J., Green S. (2011). *Cochrane Handbook for Systematic Reviews of Interventions Version 5.1.0*.

[27] Wells G. A., Shea B., O’Connell D. (2010). The Newcastle-Ottawa Scale (NOS) for assessing the quality of nonrandomised studies in meta-analyses. http://www.ohri.ca/programs/clinical_epidemiology/oxford.asp.

[28] Higgins J. P. T., Thompson S. G. (2002). Quantifying heterogeneity in a meta-analysis. *Statistics in Medicine*.

[29] Patsopoulos N. A., Evangelou E., Ioannidis J. P. (2008). Sensitivity of between-study heterogeneity in meta-analysis: proposed metrics and empirical evaluation. *International Journal of Epidemiology*.

[30] Egger M., Smith G. D., Schneider M., Minder C. (1997). Bias in meta-analysis detected by a simple, graphical test. *BMJ*.

[31] Zhao B., Luo J., Yu T., Zhou L., Lv H., Shang P. (2020). Anticancer mechanisms of metformin: a review of the current evidence. *Life Sciences*.

[32] Zhang D., Zhao Y., Wang T., Xi Y., Li N., Huang H. (2017). Diabetes mellitus and long-term mortality of ovarian cancer patients. A systematic review and meta-analysis of 12 cohort studies. *Diabetes/metabolism research and reviews*.

[33] Bae H. S., Kim H. J., Hong J. H., Lee J. K., Lee N. W., Song J. Y. (2014). Obesity and epithelial ovarian cancer survival: a systematic review and meta-analysis. *Journal of Ovarian Research*.

[34] Yang H.-S., Yoon C., Myung S.-K., Park S. M. (2011). Effect of obesity on survival of women with epithelial ovarian cancer: a systematic review and meta-analysis of observational studies. *International Journal of Gynecological Cancer*.

[35] Pu R., Shi D., Gan T. (2020). Effects of metformin in obesity treatment in different populations: a meta-analysis. *Therapeutic advances in endocrinology and metabolism*.

[36] Tsogas F. K., Majerczyk D., Hart P. C. (2021). Possible role of metformin as an immune modulator in the tumor microenvironment of ovarian cancer. *International Journal of Molecular Sciences*.

[37] Nunes M., Henriques Abreu M., Bartosch C., Ricardo S. (2020). Recycling the purpose of old drugs to treat ovarian cancer. *International Journal of Molecular Sciences*.

[38] Man X., Wang B., Tan Y., Yang X., Zhang S. (2020). Aspirin use and mortality in women with ovarian cancer: a meta-analysis. *Frontiers Oncology*.

[39] Li X., Zhou J. (2018). Impact of postdiagnostic statin use on ovarian cancer mortality: a systematic review and meta-analysis of observational studies. *British Journal of Clinical Pharmacology*.

